# Recombinant Human Proteoglycan 4 Regulates Phagocytic Activation of Monocytes and Reduces IL-1β Secretion by Urate Crystal Stimulated Gout PBMCs

**DOI:** 10.3389/fimmu.2021.771677

**Published:** 2021-12-21

**Authors:** Sandy ElSayed, Gregory D. Jay, Ralph Cabezas, Marwa Qadri, Tannin A. Schmidt, Khaled A. Elsaid

**Affiliations:** ^1^ Department of Biomedical and Pharmaceutical Sciences, Chapman University, Irvine, CA, United States; ^2^ Department of Emergency Medicine, Rhode Island Hospital, Providence, RI, United States; ^3^ Department of Pharmacology, School of Pharmacy, Jazan University, Jazan, Saudi Arabia; ^4^ Biomedical Engineering Department, University of Connecticut Health Center, Farmington, CT, United States

**Keywords:** acute gout flare, PRG4, monocytes, interleukin-1 receptor antagonist (IL-1 ra), urate crystals

## Abstract

**Objectives:**

To compare phagocytic activities of monocytes in peripheral blood mononuclear cells (PBMCs) from acute gout patients and normal subjects, examine monosodium urate monohydrate (MSU) crystal-induced IL-1β secretion ± recombinant human proteoglycan 4 (rhPRG4) or interleukin-1 receptor antagonist (IL-1RA), and study the anti-inflammatory mechanism of rhPRG4 in MSU stimulated monocytes.

**Methods:**

Acute gout PBMCs were collected from patients in the Emergency Department and normal PBMCs were obtained from a commercial source. Monocytes in PBMCs were identified by flow cytometry. PBMCs were primed with Pam3CSK4 (1μg/mL) for 24h and phagocytic activation of monocytes was determined using fluorescently labeled latex beads. MSU (200μg/mL) stimulated IL-1β secretion was determined by ELISA. Reactive oxygen species (ROS) generation in monocytes was determined fluorometrically. PBMCs were incubated with IL-1RA (250ng/mL) or rhPRG4 (200μg/mL) and bead phagocytosis by monocytes was determined. THP-1 monocytes were treated with MSU crystals ± rhPRG4 and cellular levels of NLRP3 protein, pro-IL-1β, secreted IL-1β, and activities of caspase-1 and protein phosphatase-2A (PP2A) were quantified. The peritoneal influx of inflammatory and anti-inflammatory monocytes and neutrophils in Prg4 deficient mice was studied and the impact of rhPRG4 on immune cell trafficking was assessed.

**Results:**

Enhanced phagocytic activation of gout monocytes under basal conditions (*p<0.001*) was associated with ROS generation and MSU stimulated IL-1β secretion (*p<0.05*). rhPRG4 reduced bead phagocytosis by normal and gout monocytes compared to IL-1RA and both treatments were efficacious in reducing IL-1β secretion (*p<0.05*). rhPRG4 reduced pro-IL-1β content, caspase-1 activity, conversion of pro-IL-1β to mature IL-1β and restored PP2A activity in monocytes (*p<0.05*). PP2A inhibition reversed rhPRG4’s effects on pro-IL-1β and mature IL-1β in MSU stimulated monocytes. Neutrophils accumulated in peritoneal cavities of Prg4 deficient mice (*p<0.01*) and rhPRG4 treatment reduced neutrophil accumulation and enhanced anti-inflammatory monocyte influx (*p<0.05*).

**Conclusions:**

MSU phagocytosis was higher in gout monocytes resulting in higher ROS and IL-1β secretion. rhPRG4 reduced monocyte phagocytic activation to a greater extent than IL-1RA and reduced IL-1β secretion. The anti-inflammatory activity of rhPRG4 in monocytes is partially mediated by PP2A, and *in vivo*, PRG4 plays a role in regulating the trafficking of immune cells into the site of a gout flare.

## Introduction

Gout is the most common form of crystal induced arthritis with an estimated 2012 global prevalence of 0.6% (1). The country-specific prevalence estimates vary significantly with a reported prevalence in the U.S. of 3.9%, compared to 1% or lower in China and Northern Europe (2–5). The incidence of newly diagnosed gout has more than doubled over the past decade, with greater incidences observed in males and in the later decades of life (1, 6). Hyperuricemia is the most significant risk factor for gout development where gout-associated inflammation is triggered by the deposition of poorly soluble monosodium urate monohydrate (MSU) crystals in peripheral joints and tissues (7). The most affected joints include the first metatarsophalangeal joint, and the knee, where gout often presents as an acute flare of pain and inflammation that usually resolves within one week (7, 8). Interspersed between acute gout flares are asymptomatic periods of low-grade chronic inflammation and progressive joint damage (7). Acute gout flares are treated with anti-inflammatory agents e.g., colchicine, non-steroidal anti-inflammatory drugs (NSAIDs) and corticosteroids, either alone or in combination (9, 10). None of these pharmacological agents have demonstrated superiority in controlling acute gout inflammation and their use is complicated by considerable side effects, toxicities, and relative contraindications (7, 11, 12). For patients with comorbid diabetes, renal or cardiovascular diseases, the use of interleukin-1 receptor antagonist (IL-1RA) was shown to be efficacious in the management of acute gout (13, 14). However, suboptimal clinical treatment outcomes are prevalent, with significant economic and humanistic burdens and therefore the development of novel and safer therapeutics remains an unmet clinical need.

The pathophysiology of acute gout involves the recognition of urate crystals by monocytes/macrophages in joint tissues and their subsequent phagocytosis (15, 16). Urate crystals initiate NOD-, LRR- and pyrin domain containing protein 3 (NLRP3) inflammasome activation in a mechanism that involves the generation of reactive oxygen species (ROS), resulting in recruitment of pro-caspase-1 and its conversion to active caspase-1 (16, 17). Active caspase-1 catalyzes the conversion of pro-interleukin-1 beta (pro-IL-1β) to mature IL-1β, which is the primary effector *pro*-inflammatory cytokine in gout (15, 16). Damage-associated molecular patterns (DAMPs) can also activate the NLRP3 inflammasome in gout and increase IL-1β gene expression *via* activation of toll-like receptors 2 and 4 (TLR2 and TLR4) (7, 18, 19). During an acute gout flare, circulating monocytes are recruited to the affected joint, where they contribute to inflammation *via* phagocytosis of urate crystals and differentiation into M1-like inflammatory macrophages (15, 16, 20). In response to TLR2 ligands and crystals, monocytes in peripheral blood mononuclear cells (PBMCs) from gout patients were shown to secrete higher IL-1β and other *pro*-inflammatory cytokines and chemokines compared to PBMCs from normal subjects, and the extent of IL-1β secretion was positively correlated to the annual number of acute gout attacks (21). The enhanced production of mature IL-1β by gout PBMCs was shown to likely be due to enhanced caspase-1 mediated conversion of pro-IL-1β (21). A possible explanation for the enhanced generation of mature IL-1β by gout PBMCs is chronic hyperuricemia where uric acid priming enhances IL-1β secretion by human PBMCs, *via* activating the AKT-PRAS40 autophagy pathway (22).

In our laboratory, we have previously shown that proteoglycan 4 (PRG4) and its receptor CD44, and one of several CD44 transducers, protein phosphatase-2A (PP2A) signaling axis plays a biologically significant role in regulating urate crystal inflammation. Recombinant human PRG4 (rhPRG4) inhibited urate crystal phagocytosis by murine and human macrophages, NLRP3 inflammasome activation and conversion of pro-IL-1β to mature IL-1β (23). rhPRG4 also attenuated pain and inflammation in a rat model of acute gout in the knee (23). The efficacy of rhPRG4 was likely due to its binding CD44 receptor, as CD44 was shown to mediate phagocytic uptake of urate crystals by murine and human macrophages (24, 25). The significance of the PRG4/CD44/PP2A axis was further highlighted by our recent observations that uptake of urate crystals by human monocytes was associated with a reduction in PP2A activity and that a small molecule PP2A activator reduced IL-1β secretion in human monocytes *via* attenuating pro-IL-1β production (26). While available *in vitro* and *in vivo* data suggest that rhPRG4 may be of considerable utility as a novel therapeutic in acute gout, the efficacy of rhPRG4 in gout monocytes remains unclear.

In this investigation, we aimed to quantify the *pro*-inflammatory response of normal and gout PBMCs towards combined TLR2 ligand and urate crystal stimulation with a particular focus on monocytes’ phagocytic activation and downstream IL-1β secretion. We compared the anti-inflammatory efficacy of IL-1RA and rhPRG4 and investigated how rhPRG4 regulates the activation of gout PBMCs. We also studied urate crystal stimulated THP-1 monocytes and evaluated whether rhPRG4’s effect was mediated by PP2A activity enhancement. We supplemented our *in vitro* assays with an *in vivo* peritoneal model of acute gout flare where we studied the impact of *Prg4* expression on the peritoneal influx of inflammatory classical monocytes (CMs), anti-inflammatory non-classical monocytes (NCMs) and neutrophils as well as IL-1β and CXCL1 levels (24, 27). We hypothesized that gout PBMCs display enhanced IL-1β secretion due to increased phagocytosis of urate crystals by monocytes which can be suppressed by rhPRG4, and rhPRG4’s anti-inflammatory effect is PP2A-dependent.

## Methods

### Patient Characteristics and Study Overview

Blood samples were collected from patients presenting to the Emergency Departments at Rhode Island and The Mariam Hospitals (located in Providence, RI, USA and are members of the Lifespan network) with a chief complaint of an acute gout flare. The acute gout cohort included 36 patients (28 males and 8 females) whose median age was 68 years with a range between 42 and 91 years. A total of 22 patients were on a urate-lowering therapy at the time of admission and 5 patients were using oral colchicine. PBMCs were isolated from blood samples using the Ficoll-Paque density gradient centrifugation method (28), and cells were stored in liquid nitrogen until the time of experimentation. Our study was approved by the Institutional Review Board (IRB) at Lifespan. PBMCs from twelve normal subjects were obtained from a commercial source (ATCC, USA). Monocytes in normal and gout PBMCs were identified by flow cytometry using CD14 and CD45 surface markers (29) and their phagocytic activity was determined using FITC-labeled rabbit IgG coated latex beads. Urate crystal phagocytosis by monocytes was determined by estimating the percentage of monocytes with elevated side-scatter values above a pre-determined threshold (23). Generation of reactive oxygen species (ROS) in monocytes from normal and gout PBMCs and THP-1 monocytes, following urate crystal exposure, was determined fluorometrically, and the contribution of ROS generation to IL-1β secretion by PBMCs was investigated by treating PBMCs with urate crystals ± the antioxidant N-acetylcysteine. The anti-inflammatory dose-response efficacies of IL-1RA and rhPRG4 were investigated in normal PBMCs and doses that produced maximal IL-1β attenuation by both biologics were used in follow-up experiments utilizing urate crystal stimulated gout PBMCs. The modulation of monocytes’ phagocytic activity by IL-RA and rhPRG4 was also studied in normal and gout PBMCs. To further investigate the anti-inflammatory mechanism of rhPRG4, we studied urate crystal stimulated THP-1 monocytes and assessed the impact of rhPRG4 treatment on NLRP3 protein levels, caspase-1 activation, and conversion of pro-IL-1β to mature IL-1β. Since PRG4 is a ligand of the CD44 receptor and building on our previous report that a CD44 antibody treatment activated PP2A in murine and human macrophages (24), we aimed to study the impact of rhPRG4 on PP2A activity in THP-1 monocytes. To further characterize the contribution of PP2A to rhPRG4’s mechanism of action, we evaluated the effect of co-incubating rhPRG4 with okadaic acid, a potent PP2A inhibitor (30). Normal and gout PBMCs and THP-1 monocytes were primed with a TLR2 ligand, Pam3CSK4, for 24h prior to adding urate crystals, as PBMCs and monocytes fail to secrete significant IL-1β in response to crystals alone (22, 26). Across all experiments, assays were performed with two technical replicates per experimental group, with 3-4 independent experiments for THP-1 monocytes. Our *in vivo* experiments were approved by the IACUC committee at Chapman University. We utilized the peritoneal model of acute gout flare after intraperitoneal administration of urate crystals (24). We studied the peritoneal influx of CMs (identified as CD11b+ Ly6C^hi^ CCR2+), NCMs (identified as CD11b+ Ly6C^lo^ CD43^hi^ CX3CR1+), and neutrophils (Ly6G+ Ly6B.2+) at 6h and 24h in *Prg4* gene-trap (*Prg4^GT^*) mice. We also determined IL-1β and CXLC1 levels in peritoneal lavages. The *Prg4^GT^* mouse (stock no. 025740, JAX, USA) is born lacking *Prg4* expression which can be restored *via* CRE-mediated recombination (31). We compared the peritoneal influx of immune cells in the *Prg4^GT/GT^* animals to that in *Prg4^+/+^* animals (stock # 101045, JAX; B6/129S background).

### Immunophenotyping of Monocytes and Determinations of Phagocytic Activity and ROS Generation

Normal and gout PBMCs were washed, centrifuged at 300x*g* for 10 min and seeded in RPMI 1640 media (ATCC) supplemented with 2% heat-inactivated fetal bovine serum (FBS) and 1% penicillin-Streptomycin (Sigma Aldrich, USA) at a density of 1.0 x 10^6^ cells per well. Culture media was supplemented with 50 IU benzonase (Sigma Aldrich) to avoid cell clumping. Human THP-1 leukemic monocytic cells (ATCC) were seeded as described above. Immunophenotyping of monocytes in PBMCs was performed by flow cytometry using APC/Cyanine7 anti-human CD14 antibody (monocyte marker; 1:20 dilution) (BioLegend, USA) and PE-anti human CD45 antibody (pan-leukocyte marker; 1:25 dilution) (BioLegend). Following treatments, PBMCs were collected and centrifuged at 3,000 rpm for 5 min. Cells were then incubated with Zombie Violet viability dye in FACS blocking buffer (0.5% BSA, 2% FBS in PBS) for 10 min on ice. Subsequently, cells were stained with anti-CD14 and anti-CD45 antibodies in FACS staining/washing buffer (0.5% BSA, 0.05% sodium azide in PBS) for 20 min. Cells were then washed twice with FACS washing buffer followed by flow cytometric analysis (BD FACSAria). Flow cytometry plots and analyses were generated using Flow Jo^®^ software (BD Biosciences, USA), and positive staining thresholds were determined using fluorescence minus one (FMO) control.

Phagocytic activity of monocytes was performed using FITC-labeled rabbit IgG-coated latex beads (Cayman Chemicals, USA). PBMCs or THP-1 monocytes were incubated with Pam3CSK4 (1μg/mL) (Invivogen, USA) for 24h. Subsequently, fluorescently labeled beads were co-incubated with cells for 4h and the fluorescence intensity (FI) geometric means of bead-positive cells were calculated. Bead-positive cells were determined using an FMO control.

ROS generation in monocytes of normal and gout PBMCs and THP-1 monocytes was determined using the DCFDA/H2DCFDA cellular ROS Assay Kit (Abcam, USA). The principle of the assay is based on the oxidation of a non-fluorescent probe by cellular hydroxyl, peroxyl and other ROS species into a fluorescent product. PBMCs were incubated with DCFDA/H2DCFDA for 30 min at 37°C followed by incubation with MSU crystals (200μg/mL) (Invivogen) for 2h. Subsequently, PBMCs were collected and stained with anti-CD14 and anti-CD45 antibodies as described above to identify monocytes. Quantitation of ROS generation in monocytes was performed by initially gating for AlexFluor-488 positive monocytes and then calculating the FI geometric means of this population. Supernatant mature IL-1β levels were also measured at 2h using an ELISA kit (R&D Systems, USA).

### Dose Response and Anti-Inflammatory Efficacies of IL-1RA and rhPRG4 in Urate Crystal Treated Normal and Gout PBMCs

Normal PBMCs were stimulated with Pam3CSK4 (1μg/mL) ± recombinant human IL-1RA (GenScript, USA; MW ~ 25 kDa) (100, 250, 500 ng/mL, 1 and 2μg/mL) or rhPRG4 (Lubris, USA; apparent MW ~ 460 kDa and ~ 1M Da as disulfide bonded dimer) (10, 50, 100 and 200μg/mL) (32) for 24h followed by MSU crystals (200μg/mL) for 6h and secreted IL-1β levels were determined by ELISA (R&D Systems). Gout PBMCs were stimulated with Pam3CSK4 (1μg/mL) ± IL-1RA (250ng/mL) or rhPRG4 (200μg/mL) for 24h followed by adding urate crystals and measurement of secreted IL-1β levels as described above. The efficacy of IL-1RA (250ng/mL) or rhPRG4 (200μg/mL) in modulating the extent of bead phagocytosis by normal and gout monocytes was determined as described above following Pam3CSK4 (1μg/mL) priming ± IL-1RA or rhPRG4 for 24h.

### Impact of N-Acetylcysteine (NAC) Treatment on ROS Generation in Normal PBMCs and Related IL-1β Secretion

Normal PBMCs were primed with Pam3CSK4 (1μg/mL) for 24h followed by treatment with MSU crystals (200μg/mL) ± N-acetylcysteine (20 mM) (Sigma Aldrich) and ROS generation and secreted IL-1β levels were determined as described above at 2h and 6h, *respectively*. In these ROS experiment, we used 20μM DCFDA while in the gout and normal monocyte ROS experiments, we used 10μM DCFDA. THP-1 monocyte ROS experiments were performed as described above using 20μM DCFDA.

### Impact of IL-1RA or rhPRG4 Treatments on IL-1β Gene Expression, Intracellular Pro-IL-1β and Secreted IL-1β in Urate Crystal Stimulated THP-1 Monocytes

THP-1 monocytes were treated with Pam3CSK4 (1μg/mL) for 24h followed by MSU crystals (200μg/mL) ± IL-1RA (250ng/mL) or rhPRG4 (200μg/mL) for 6h. IL-1β gene expression was performed as previously described (26), using commercially available primers and probes for IL-1β (Hs01555410_m1) and β-actin (Hs00194899_m1) (ThermoFisher Scientific, USA), and the cycle threshold (Ct) value of IL-1β was normalized to the Ct value of β-actin in the same sample, and the relative expression in the different experimental groups compared to untreated controls was computed using the 2^-ΔΔCt^ method (26). In another set of experiments, cells were treated and subsequently collected and lysed using RIPA buffer + 1% protease inhibitor (ThermoFisher Scientific), and cell lysate total protein was determined using the micro-BCA assay kit (Sigma Aldrich). Pro-IL-1β levels in cell lysates (MyBioSource, USA) and supernatant mature IL-1β levels were determined by ELISA and analyte concentrations in cell lysates were normalized to total protein.

### Role of PP2A in Mediating rhPRG4’s Anti-Inflammatory Efficacy in Urate Crystal Stimulated Human THP-1 Monocytes

PP2A was immunoprecipitated from THP-1 monocyte cell lysates following Pam3CSK4 priming for 24h and MSU crystals (200μg/mL) ± IL-1RA (250ng/mL) or rhPRG4 (200μg/mL) for 6h. PP2A activity was determined as previously described (26), and normalized to cell lysate total protein. To further investigate the role of PP2A in mediating rhPRG4’s effect, THP-1 monocytes were stimulated with Pam3CSK4 for 24h followed by MSU crystals (200μg/mL) ± rhPRG4 (200μg/mL) ± okadaic acid (5nM) (Cayman Chemicals) for 6h. Intracellular pro-IL-1β, NLRP3 protein (MyBioSource) and secreted IL-1β levels were determined as described above. To determine caspase-1 activity in THP-1 monocytes, 500,000 cells were seeded in black 96-well plate with clear bottoms (Sigma Aldrich) and stimulated as described above. Caspase-1 activity was determined using the cell based active capase-1 staining kit (Abcam).

### *In Vivo* Peritoneal Model of Acute Gout Flare in *Prg4^GT/GT^* and *Prg4^+/+^* Mice and Impact of rhPRG4 Treatment on Peritoneal Influx of Monocytes and Neutrophils and Peritoneal Lavage IL-1β and CXCL1 Levels

We have utilized the murine peritoneal model of acute gout as previously described (24), and compared the influx of pro-inflammatory CMs, anti-inflammatory NCMs and neutrophils at 6h and 24h following intra-peritoneal administration of MSU crystals (2mg in 200μL) in *Prg4^GT/GT^* and *Prg4^+/+^* mice. Animals were 2-3 months old and included equal numbers of males and females (n=4 in each group at each time point). Lavaging was performed by injecting 3 mL of cold PBS into the peritoneal cavity followed by shaking for 30–60 seconds and lavage aspiration. Lavage fluids were centrifuged at 450 g for 10 min and cell pellets were resuspended in 1 mL PBS and subjected to immunophenotyping, as described above, while supernatants were used for ELISAs. The following fluorochrome-conjugated antibodies were used at manufacturer’s recommended dilutions: APC-Cy7-anti-CD11b, Alexa-488-anti-Ly6C, PE-anti-CCR2, APC-anti-CD43, PerCP-Cy5.5-anti-CX3CR1, APC-Cy7-anti-Ly6G (BioLegend) and Alexa-488-anti-Ly6B.2 (Fisher Scientific). Immunophenotyping for CMs and NCMs was performed independently of neutrophil immunophenotyping, and the number of cells in populations of interest were estimated using Precision Counting Beads (BioLegend). In another set of experiments, rhPRG4 (50μL; 1 mg/mL) or PBS (50μL) were administered at 6h following urate crystal administration and peritoneal lavaging and immunophenotyping were performed at 24h. IL-1β and CXCL1 levels were determined by ELISA (R&D Systems).

### Statistical Analyses

Continuous variables were initially evaluated to determine if they satisfy the requirements of parametric statistical tests. Statistical comparisons between two groups were performed using Student’s *t*-test or the non-parametric Mann-Whitney U test. Statistical comparisons of multiple groups were performed using one-way and two-way analysis of variance (ANOVA) followed by Tukey’s *post*-hoc test for parametric data or the equivalent ANOVA on the ranks for non-parametric data. A *p* value of < 0.05 was considered statistically significant. Data are graphically represented as scatter plot bar graphs with mean ± standard deviation indicated.

## Results

### Monocytes From Gout Patients Demonstrated Enhanced Basal Bead Uptake and Elevated IL-1β Secretion in Response to TLR2 Ligand Priming and Urate Crystal Exposure

**Figure 1:**
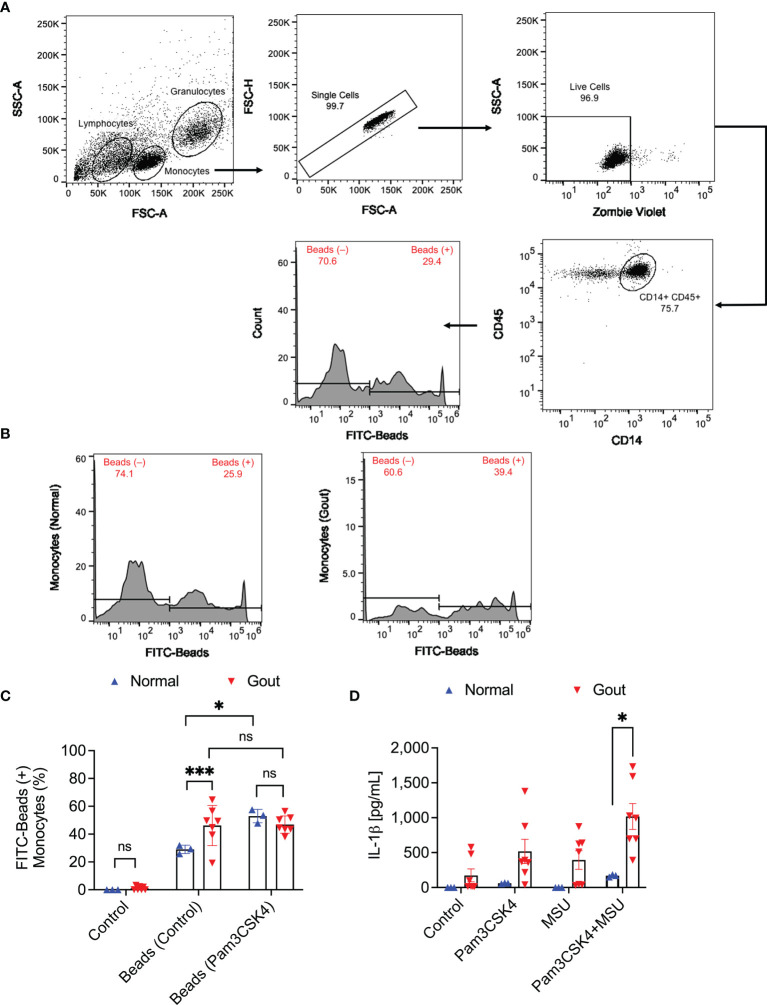
Analysis of the phagocytic activity of monocytes in peripheral blood mononuclear cells (PBMCs) of normal subjects (n=3) and gout patients (n=7) and its relationship to interleukin-1 beta (IL-1β) release in response to priming with Pam3CSK4, a toll-like receptor 2 ligand, and treatment with monosodium urate monohydrate (MSU) crystals. To assess phagocytic activation, PBMCs were treated with Pam3CSK4 (1μg/mL) for 24h followed by co-incubation with FITC-labeled rabbit IgG-coated latex beads and determination of percent bead-positive monocytes. Alternatively, PBMCs were incubated with MSU crystals (200μg/mL) and secreted IL-1β levels were determined by ELISA at 6h. Analysis of FITC-bead positive monocytes was performed using 2-way ANOVA followed by Tukey’s *post*-hoc test, and analysis of IL-1β levels was performed by multiple *t*-tests followed by a *post*-hoc false discovery rate analysis using two stage step-up approach. ns, non-significant; *p < 0.05; ***p < 0.001. **(A)** Flow cytometry gating strategy for monocytes in PBMCs. Identification of monocytes was conducted using the expected forward and side scatter (FSC-A and SSC-A) ranges and single cells were gated using FSC-A and FSC-H. Viable cells were identified using Zombie Violet viability dye and monocytes were confirmed using CD14 and CD45 surface markers. Subsequently, bead positive monocytes were gated based on thresholds established using fluorescence minus one control. **(B)** Representative flow cytometry histograms depicting a higher percentage of bead-positive monocytes in a gout patient compared to a normal subject. **(C)** Monocytes of gout patients had higher basal phagocytic activities compared to normal subjects. TLR2 ligand priming increased phagocytic activities of normal subjects’ monocytes but not gout patients and phagocytic activities in primed samples were not different between normal and gout subjects. **(D)** A combination of TLR2 ligand priming and MSU crystal challenge resulted in higher secreted IL-1β levels from gout patients’ PBMCs.

The flow cytometry gating strategy to identify monocytes in PBMCs and quantitation of FITC-labeled beads’ uptake by monocytes are shown in **Figure 1A**. We initially identified monocytes using a combination of side scatter area (SSC-A) and forward scatter area (FSC-A) values (29). Singlet cells were subsequently identified based on FSC-A and FSC-height (FSC-H) and monocytes were confirmed by dual positive staining for CD14 and CD45 markers. Monocytes’ phagocytic activity was determined by estimating the percentage of monocytes positive for FITC-labeled beads. Representative flow cytometry plots showing enhanced bead uptake in gout monocytes are shown in **Figure 1B**. We observed that uptake of FITC-labeled beads by gout monocytes under basal condition was higher than their uptake by normal monocytes (*p<0.001*; **Figure 1C**). Under TLR2 stimulated condition, bead phagocytosis by normal monocytes increased (*p<0.05*) while gout monocytes showed no significant change in their bead uptake (*p>0.05*). In stimulated samples, bead phagocytosis was not different between normal and gout monocytes (*p>0.05*). The enhanced basal phagocytic activity of gout monocytes indicates that these cells may have already been primed towards urate crystal uptake, potentially due to the systemic inflammatory environment in acute gout. In contrast, normal subjects’ monocytes had to be primed with a TLR2 ligand to increase their phagocytic activity, to a level comparable to that of gout monocytes at baseline. A combination of TLR2 ligand and urate crystals resulted in greater IL-1β secretion from gout PBMCs compared to normal PBMCs (*p<0.05*; **Figure 1D**).

### IL-1RA and rhPRG4 Displayed Dose-Dependent Reductions in IL-1β Secretion From Normal PBMCs and the Anti-Inflammatory Effect of Both Treatments Was Related to Reducing Phagocytic Activation of Normal Monocytes

**Figure 2:**
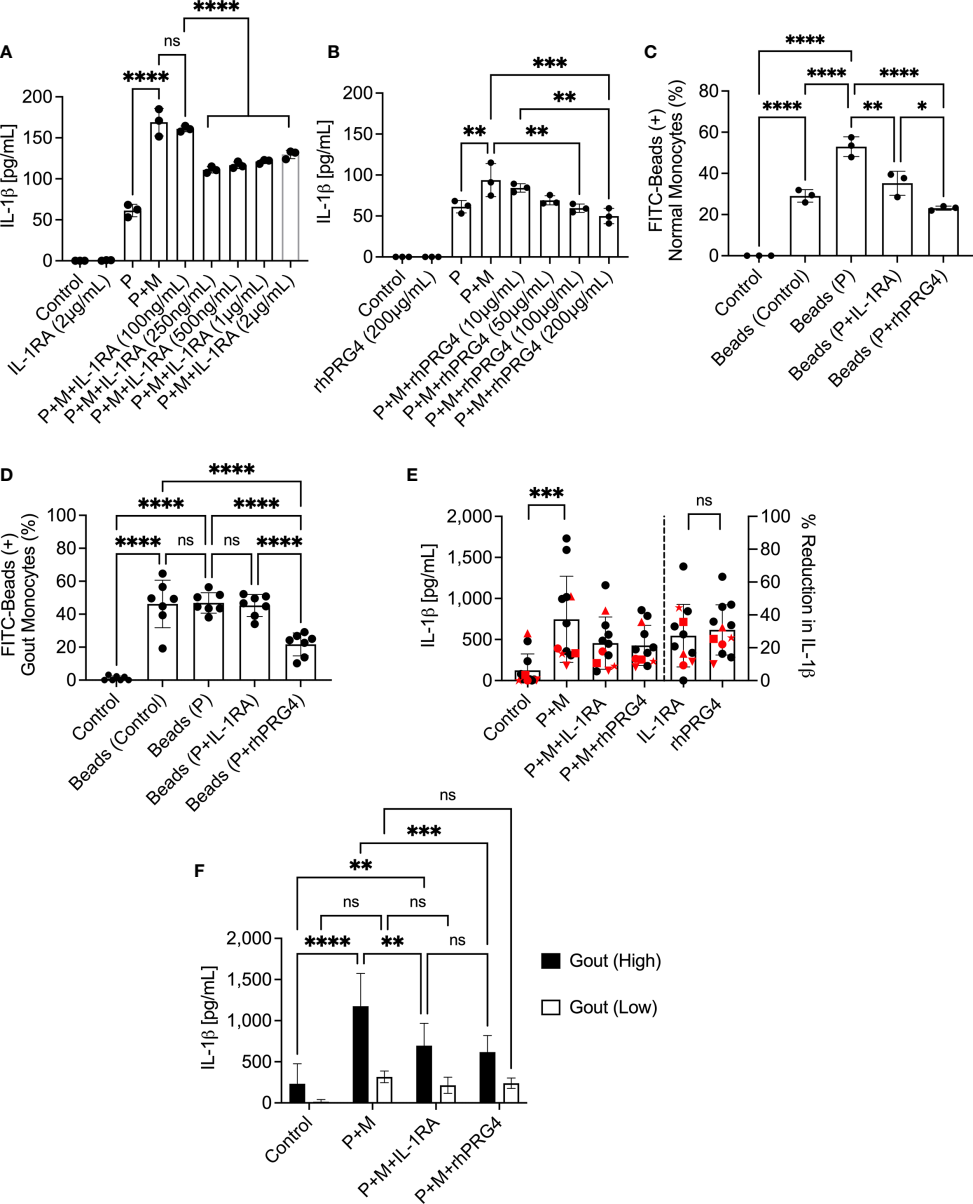
Dose-response of interleukin-1 receptor antagonist (IL-1RA) and recombinant human proteoglycan 4 (rhPRG4) in Pam3CSK4 (P), a toll-like receptor 2 ligand, and monosodium urate monohydrate (M) crystal-stimulated peripheral blood mononuclear cells (PBMCs) of normal subjects and efficacy of IL-1RA and rhPRG4 in modulating phagocytic activation of monocytes of normal subjects (n=3) and gout patients (n=7 to 12) and secretion of mature interleukin-1 beta (IL-1β) by urate crystal-stimulated gout PBMCs. To assess phagocytic activation, PBMCs were treated with Pam3CSK4 (1μg/mL) for 24h followed by co-incubation with FITC-labeled rabbit IgG-coated latex beads and determination of percent bead-positive monocytes, using the gating strategy shown in **Figure 1A**. Alternatively, PBMCs were incubated with urate crystals (200μg/mL) and secreted IL-1β levels were determined by ELISA. Gout PBMCs were categorized, based on urate crystal-induced IL-1β secretion, as either high IL-1β secreting (IL-1β concentrations > 500pg/mL) (n=6) or low IL-1β secreting (IL-1β concentrations < 500pg/mL) (n=6) and the differential efficacies of IL-1RA and rhPRG4 on both populations was investigated. Statistical analyses included one and two-way ANOVAs followed by Tukey’s *post*-hoc test. ns, non-significant; *p < 0.05; **p < 0.01; ***p < 0.001; ****p < 0.0001. **(A)** IL-1RA (250ng/mL and higher) reduced IL-1β secretion by PBMCs of normal subjects. **(B)** rhPRG4 (100&200μg/mL) reduced IL-1β secretion by PBMCs of normal subjects. **(C)** IL-1RA (250ng/mL) and rhPRG4 (200μg/mL) reduced phagocytic activation of monocytes in PBMCs of normal subjects. rhPRG4 treatment reduced bead phagocytosis by normal monocytes compared to IL-1RA. **(D)** rhPRG4 (200μg/mL) reduced bead phagocytosis by gout monocytes, while IL-1RA (250ng/mL) did not alter fluorescent bead uptake by the same monocytes. **(E)** IL-1RA (250ng/mL) and rhPRG4 (200μg/mL) reduced IL-1β secretion by gout PBMCs by similar magnitudes. PBMCs from patients receiving colchicine (n=5) are highlighted in red. 4 out of 5 samples are classified as low-IL-1β secreting gout PBMCs. **(F)** IL-1RA (250ng/mL) and rhPRG4 (200μg/mL) reduced IL-1β secretion from high-IL-1β secreting gout PBMCs but not from low-IL-1β secreting PBMCs.

IL-1RA treatment reduced IL-1β secretion by MSU stimulated normal PBMCs with efficacy observed at 250 ng/mL and higher (*p<0.0001*; **Figure 2A**), whereby the anti-inflammatory efficacy of IL-1RA did not change above the 250 ng/mL level. Meanwhile, rhPRG4 reduced IL-1β secretion in the same model at 100 and 200 μg/mL (*p<0.01*; *p<0.001*; **Figure 2B**), with the 200 μg/mL concentration appearing to produce marginally better reduction in IL-1β secretion compared to the 100 μg/mL concentration. The magnitudes of IL-1β secretion from positive control groups were different between the IL-1RA and rhPRG4 experiments, with approximately 30% less IL-1β secretion in the rhPRG4 dose-response experiment. This might be attributed to the different normal PBMCs specimens used between the two experiments. Nonetheless, IL-1RA and rhPRG4 produced robust maximal reductions in IL-1β secretions. At dose levels that maximally reduced IL-1β secretion, IL-1RA (250 ng/mL) and rhPRG4 (200 μg/mL) reduced normal monocytes’ phagocytic activity following TLR2 ligand treatment (*p<0.01*; *p<0.0001*; **Figure 2C**) but rhPRG4 displayed a greater reduction in FITC-labeled beads’ uptake by normal monocytes compared to IL-1RA (*p<0.05*).

### rhPRG4 But Not IL-1RA Reduced Basal and TLR2 Ligand Stimulated Bead Uptake by Gout Monocytes and the Anti-Inflammatory Effects of Both IL-1RA and rhPRG4 Were Dependent on the Magnitude of IL-1β Secretion by Gout PBMCs

rhPRG4 (200 μg/mL) reduced basal and TLR2 stimulated bead uptake by gout monocytes compared to IL-1RA (250 ng/mL) treatment (*p<0.0001* for both comparisons; **Figure 2D**). We observed high variability in the anti-inflammatory effects of IL-1RA and rhPRG4 in urate crystal stimulated gout PBMCs (**Figure 2E**). The mean reduction in IL-1β (95% CI) in the gout cohort was 27.3% (15.2%-39.4%) with IL-1RA and 30.9% (21.1%-40.6%) with rhPRG4. PBMCs from patients on oral colchicine had blunted IL-1β secretion, as 4 out of 5 specimens (all 5 specimens are highlighted in red) were in the low IL-1β secreting group (**Figure 2E**, left panel). In addition, percentage reductions in IL-1β secretion by IL-1RA or rhPRG4 in the majority of these specimens were lower than the average for the entire cohort (**Figure 2E**, right panel). IL-1RA and rhPRG4 treatments reduced IL-1β secretion by gout PBMC specimens that exhibited high urate crystal stimulated IL-1β release (>500 pg/mL) (*p<0.01*; *p<0.001*; **Figure 2F**). In gout PBMCs with IL-1β secretion < 500 pg/mL, IL-1RA and rhPRG4 treatments showed no significant reductions in IL-1β release (*p>0.05*). These findings argue that rhPRG4 exerts its anti-inflammatory effect by virtue of reducing monocytes’ phagocytic activation and thus their ability to internalize urate crystals and that the greater the stimulation of monocytes by urate crystals, the more significant is the anti-inflammatory effect of rhPRG4.

### Enhanced Urate Crystal Uptake by Gout Monocytes Elicited Higher ROS Levels and NAC Treatment Reduced IL-1β Secretion in Urate Crystal Stimulated Normal PBMCs

**Figure 3:**
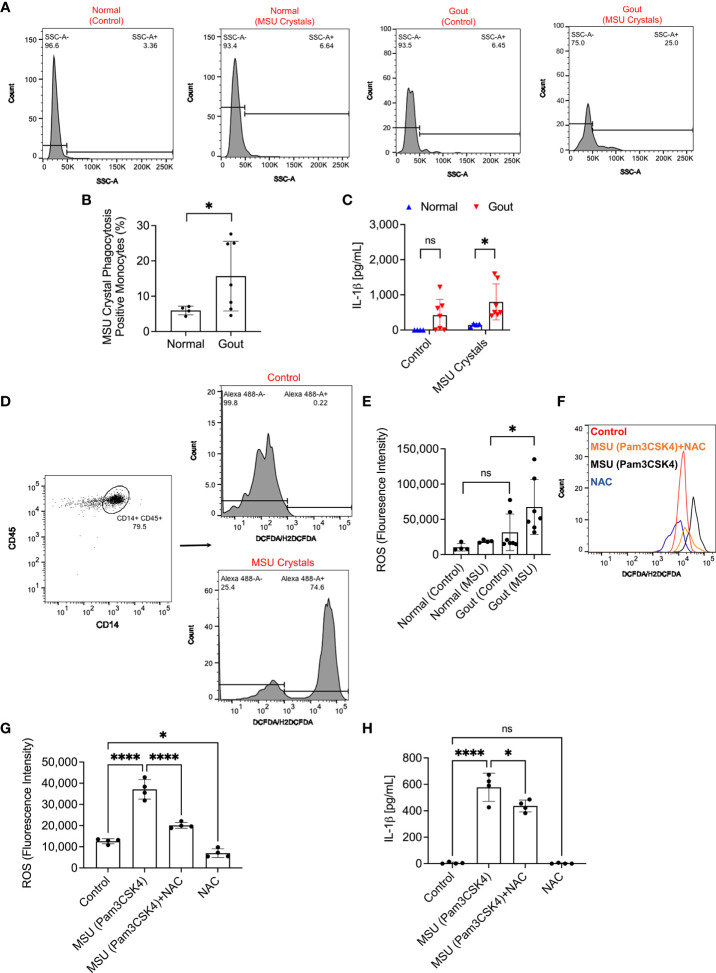
Analysis of monosodium urate monohydrate (MSU) crystal phagocytosis and its relationship to reactive oxygen species (ROS) generation in monocytes of peripheral blood mononuclear cells (PBMCs) of normal subjects (n=3) and gout patients (n=7) and secretion of interleukin-1 beta (IL-1β). PBMCs were primed with Pam3CSK4 (1μg/mL) for 24h followed by MSU crystals (200μg/mL) and MSU crystal phagocytosis was determined by assessing the change in monocytes’ side scatter (SSC-A) profile and mature IL-1β levels were determined by ELISA at 2h. ROS generation in monocytes was determined using DCFDA/H2DCFDA probe, and geometric means of fluorescence intensities (FI) of Alexa Fluor 488-positive monocytes were calculated and compared across groups. To further delineate the role of ROS in IL-1β secretion, PBMCs of normal subjects (n=4) were primed with Pam3CSK4 (1μg/mL) for 24h followed by MSU crystals (200μg/mL) ± N-acetylcysteine (NAC) (20 mM). Statistical analyses included Student’s *t*-test and one-way ANOVA followed by Tukey’s *post*-hoc test. ns, non-significant; *p < 0.05; ****p < 0.0001. **(A)** Representative flow cytometry histograms showing increased monocytes with SSC-A values above a pre-determined threshold indicative of MSU crystal phagocytosis. **(B)** Phagocytosis of MSU crystals by gout monocytes was higher than normal monocytes. **(C)** IL-1β secretion from MSU-challenged gout PBMCs was higher than normal PBMCs. **(D)** Representative flow cytometry histograms of DCFDA/H2DCFDA stained normal monocytes at baseline and following MSU crystal incubation. Monocytes with Alexa Fluor 488 FI values above 1.0 x 10^3^ were considered positive and the FI geometric mean of this population was determined. Gating of monocytes was performed as shown in **Figure 1A**. **(E)** ROS generation was higher in gout monocytes compared to normal monocytes. **(F)** Representative flow cytometry histogram showing an increase in ROS generation in monocytes as a result of MSU crystal exposure compared to control, which was attenuated with NAC treatment. **(G)** NAC treatment reduced ROS generation in monocytes of MSU crystal challenged PBMCs. **(H)** NAC treatment reduced IL-1β release by MSU challenged PBMCs.

We identified monocytes with urate crystal uptake according to changes in SSC-A values as shown in representative flow cytometry histograms in **Figure 3A**. In gout monocytes, we detected higher crystal positive fractions compared to normal monocytes (*p<0.05*; **Figure 3B**). The enhanced urate crystal phagocytosis by gout monocytes was associated with a significant increase in secreted IL-1β levels, at 2h, by gout PBMCs compared to normal PBMCs (*p<0.05*; **Figure 3C**). The gating strategy to quantify ROS generation in normal and gout monocytes is shown in **Figure 3D**. Monocytes were gated according to the strategy presented in **Figure 1A** and the percentage of monocytes positive for ROS was quantified. The positivity threshold was determined using control untreated samples, and the FI geometric means in ROS-positive monocytes were determined and compared across treatment groups. Urate crystals did not appreciably increase ROS levels in normal monocytes (*p>0.05*; **Figure 3E**), which might be due to the quantity of urate crystals vis-à-vis the number of monocytes in the PBMC specimen, as well as the concentration of the DCFDA reagent used. In contrast, ROS levels in urate crystal stimulated gout monocytes were higher than corresponding levels in normal monocytes (*p<0.05*). Collectively, our findings argue that the higher secreted IL-1β levels in gout PBMCs were associated with increased ROS levels in monocytes, potentially due to increased urate crystal uptake. To further evaluate the significance of ROS generation in urate crystal stimulated monocytes in the context of IL-1β secretion, we neutralized ROS using NAC. Representative flow cytometry histograms showing attenuated ROS signal in TLR2 ligand primed and urate crystal stimulated normal monocytes with NAC are presented in **Figure 3F**. We optimized the staining protocol for normal PBMCs to detect a positive ROS signal with TLR2 ligand priming and MSU crystals at 2h. Gating of monocytes was performed as shown in **Figures 1A**, **3A**. NAC treatment reduced basal and urate crystal stimulated ROS levels (*p<0.05*; *p<0.0001*; **Figure 3G**). The reduction in ROS levels resulted in a reduction in secreted IL-1β levels (*p<0.05*; **Figure 3H**). This indicates that ROS generation plays a causal role in IL-1β secretion by urate crystal stimulated monocytes.

### rhPRG4 Reduced TLR2 Ligand Stimulated Bead Uptake, ROS Generation, Intracellular Pro-IL-1β and Secreted Mature IL-1β Levels and Enhanced PP2A Activity in Human THP-1 Monocytes

**Figure 4:**
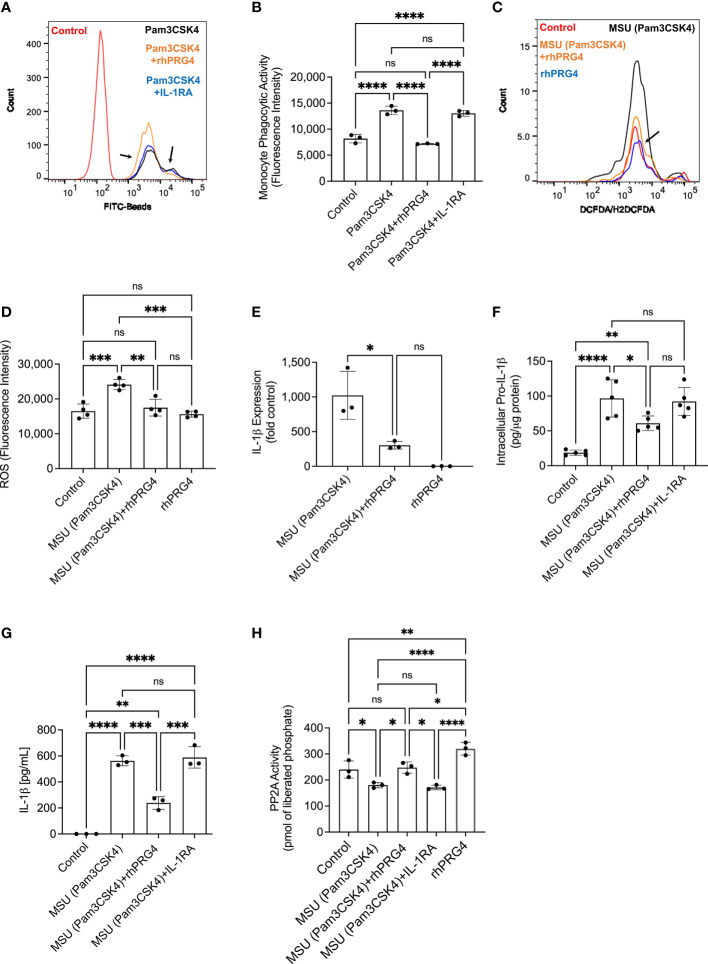
Impact of recombinant human proteoglycan-4 (rhPRG4) or interleukin-1 receptor antagonist (IL-1RA) treatments on phagocytic activation of human THP-1 monocytes, intracellular pro interleukin-1 beta (pro-IL-1β), secreted mature IL-1β and activation of NLRP3 in response to monosodium urate monohydrate (MSU) crystal challenge and role of protein phosphatase-2A (PP2A) in mediating rhPRG4’s effect. Reactive oxygen species (ROS) generation in THP-1 monocytes ± rhPRG4 was determined using the DCFDA/H2DCFDA probe, and geometric means of fluorescence intensities (FI) of Alexa Fluor 488-positive cells were calculated and compared across groups at 2h post treatments. THP-1 monocytes were primed with Pam3CSK4 (1μg/mL) for 24h ± rhPRG4 (200μg/mL) or IL-1RA (250ng/mL) followed by co-incubation with FITC-beads for 2h and THP-1 phagocytic activation was determined as shown in **Figure 1A**. Alternatively, THP-1 monocytes were challenged with MSU crystals (200μg/mL) for 6h ± rhPRG4 (200μg/mL) or IL-1RA (250ng/mL) followed by analysis of IL-1β gene expression, intracellular pro-IL-1β and secreted mature IL-1β by ELISA and PP2A activity following PP2A immunoprecipitation. Intracellular pro-IL-1β and PP2A activity were normalized to total isolated protein. Statistical analysis was performed using one-way ANOVA followed by Tukey’s *post*-hoc test. ns, non-significant; *p < 0.05; **p < 0.01; ***p < 0.001; ****p < 0.0001. **(A)** Representative flow cytometry histograms showing enhanced phagocytosis of FITC labeled beads by TLR2 ligand primed THP-1 monocytes and rhPRG4 treatment appeared to reduce phagocytosis of FITC-labeled beads (shown by arrows). **(B)** rhPRG4 treatment reduced FITC-labeled beads’ phagocytosis by THP-1 monocytes. **(C)** Representative flow cytometry histograms showing reduced ROS fluorescence intensity with rhPRG4 treatment (shown by an arrow). **(D)** rhPRG4 treatment reduced ROS generation in THP-1 monocytes. **(E)** rhPRG4 reduced IL-1β gene expression in THP-1 monocytes. **(F)** rhPRG4 reduced pro-IL-1β levels in THP-1 monocytes. **(G)** rhPRG4 reduced mature IL-1β secreted by THP-1 monocytes. **(H)** rhPRG4 treatment increased PP2A activity in MSU challenged THP-1 monocytes.

Representative flow cytometry histograms depicting uptake of FITC-labeled beads by TLR2 ligand primed THP-1 monocytes ± IL-1RA (250 ng/mL) or rhPRG4 (200 μg/mL) are presented in **Figure 4A**. rhPRG4 treatment reduced uptake of FITC-labeled beads by THP-1 monocytes (*p<0.0001*; **Figure 4B**). In contrast, IL-1RA treatment did not alter bead uptake by THP-1 monocytes (*p>0.05*). Representative flow cytometry histograms showing qualitatively blunted ROS generation in rhPRG4-treated THP-1 monocytes are shown in **Figure 4C**. Quantitatively, FI geometric means of rhPRG4-treated monocytes were reduced (*p<0.01*; **Figure 4D**), supporting a significant reduction in urate crystal associated ROS generation with rhPRG4 treatment. rhPRG4 reduced IL-1β gene expression (*p<0.05*; **Figure 4E**). In addition, rhPRG4 reduced intracellular pro-IL-1β (*p<0.05*; **Figure 4F**) and secreted IL-1β *(p<0.001*; **Figure 4G**) levels in urate crystal stimulated THP-1 monocytes. Urate crystals reduced PP2A activity in THP-1 monocytes (*p<0.05*; **Figure 4H**), and rhPRG4 treatment restored PP2A activity to basal levels (*p<0.05*; **Figure 4H**). In summary, the reduction in monocytic phagocytic activity by rhPRG4 was associated with a reduction in ROS generation, IL-1β expression, pro-IL-1β production and ultimately secretion of mature IL-1β. The phagocytosis of urate crystals was associated with a reduction in PP2A activity which was reversed with rhPRG4. Since ROS generation plays a causal role in IL-1β secretion by urate crystal stimulated monocytes, rhPRG4’s anti-inflammatory mechanism appears to be mediated by reducing ROS generation, subsequent to reducing monocyte phagocytic activation.

### The Anti-Inflammatory Effect of rhPRG4 in Urate Crystal Stimulated THP-1 Monocytes Is Partially Mediated by PP2A

**Figure 5:**
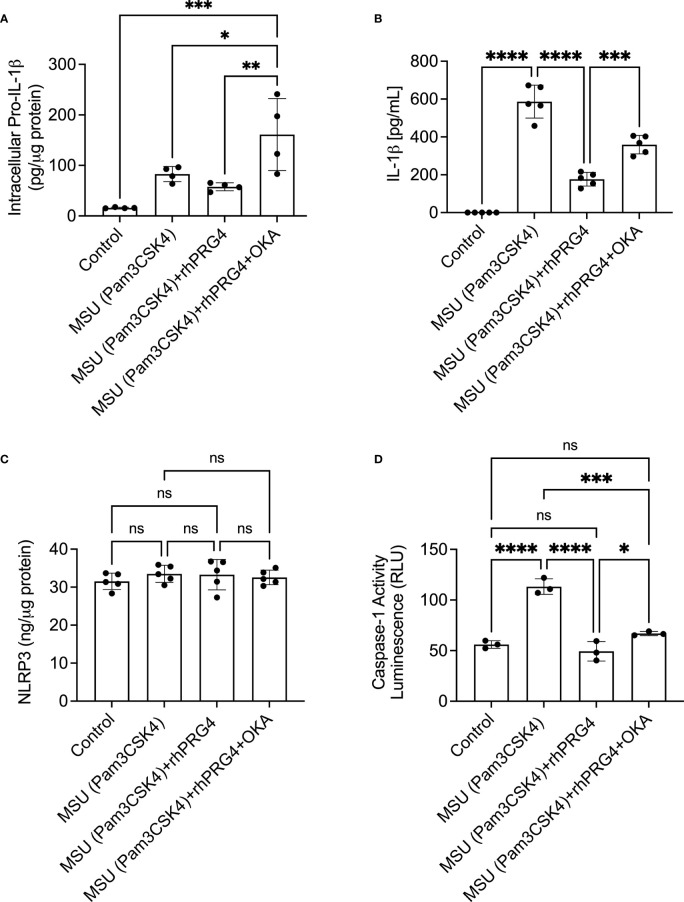
Role of protein phosphatase-2A (PP2A) in mediating rhPRG4’s anti-inflammatory effect in monosodium urate monohydrate (MSU) crystals challenged THP-1 human monocytes. THP-1 monocytes were primed with Pam3CSK4 (1μg/mL) for 24h followed by MSU (200μg/mL) crystals ± rhPRG4 (200μg/mL) ± okadaic acid (OKA) (5nM) for 6h and intracellular pro-interleukin-1 beta (pro-IL-1β), secreted mature IL-1β, NLRP3 protein, and caspase-1 activity were quantified. Intracellular pro-IL-1β, NLRP3 and caspase-1 activity were normalized to total isolated protein. Statistical analysis was performed using one-way ANOVA followed by Tukey’s *post*-hoc test. ns, non-significant; *p < 0.05; **p < 0.01; ***p < 0.001; ****p < 0.0001. **(A)** OKA co-treatment increased pro-IL-1β content in rhPRG4-treated THP-1 monocytes. **(B)** OKA co-treatment increased secreted IL-1β levels in rhPRG4-treated THP-1 monocytes. **(C)** NLRP3 protein content did not change as a result of rhPRG4 ± OKA treatments. **(D)** OKA co-treatment increased caspase-1 activity in rhPRG4-treated THP-1 monocytes.

Okadaic acid and rhPRG4 co-treatment increased intracellular pro-IL-1β levels compared to rhPRG4 treatment alone (*p<0.01*; **Figure 5A**). Similarly, okadaic acid co-treatment increased secreted IL-1β levels (*p<0.001*; **Figure 5B**) in urate crystal stimulated THP-1 monocytes. NLRP3 protein levels in THP-1 monocytes did not change with urate crystals ± rhPRG4 ± okadaic acid treatments (*p>0.05* for all comparisons; **Figure 5C**). Casepase-1 activity in THP-1 monocytes increased following urate crystal exposure, which was reversed with rhPRG4 treatment (*p<0.0001*; **Figure 5D**). PP2A activity enhancement appeared to contribute to rhPRG4’s inhibition of capsase-1 activity as okadaic acid co-treatment partially reversed rhPRG4’s effect (*p<0.05*). These results support the conclusion that rhPRG4’s biological effects in urate crystal stimulated THP-1 monocytes were partially mediated by PP2A, where PP2A activation was seen with rhPRG4 addition and inhibition of PP2A activity significantly reduced rhPRG4’s anti-inflammatory effect.

### Lack of Effective Resolution of Gout Inflammation Was Observed in *Prg4^GT/GT^* Animals, and rhPRG4 Treatment Increased Anti-Inflammatory NCMs Influx and Reduced Neutrophil Accumulation in the Peritoneal Acute Gout Flare Model

**Figure 6:**
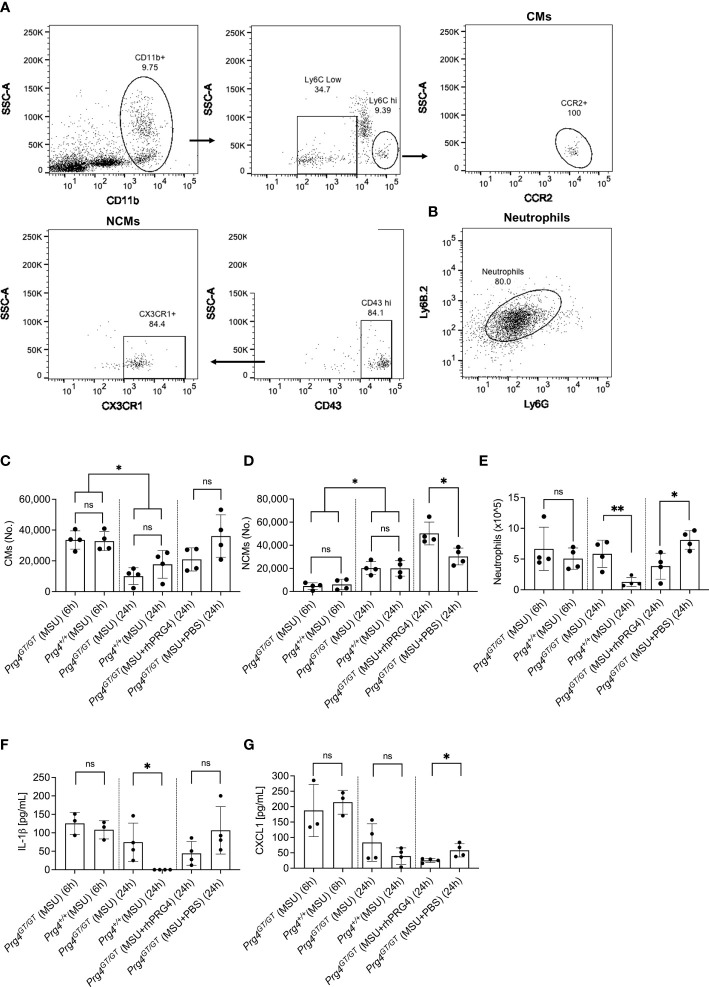
Recruitment of inflammatory classical monocytes (CMs), anti-inflammatory nonclassical monocytes (NCMs) and neutrophils in monosodium urate (MSU) crystal induced peritoneal inflammation model in proteoglycan 4 (Prg4) gene-trap (*Prg4^GT/GT^*) and Prg4 competent (*Prg4^+/+^*) mice. Peritoneal lavages (PLs) were collected at 6h and 24h. CMs were identified as CD11b+ Ly6C^hi^ CCR2+ and NCMs were identified as CD11b+ Ly6C^lo^ CD43^hi^ CX3CR1+. Neutrophils were identified using Ly6G and Ly6B.2 surface markers. PL cell populations of interest were determined using Precision Counting Beads. PL IL-1β and CXCL1 levels were determined by ELISAs. Recombinant human PRG4 (rhPRG4) (50μL; 1 mg/mL) or PBS (50μL) were administered intra-peritoneally at 6h following MSU crystal injection in the *Prg4^GT/GT^* mice with PLs collected at 24h. We utilized 4 animals in each group at each time point balanced between males and females with an age range of 2-3 months. Statistical analyses included Student’s *t*-test and two-way ANOVA followed by Tukey’s *post*-hoc test. ns, non-significant; *p < 0.05; **p < 0.01. **(A)** Flow cytometry gating strategy to identify inflammatory CMs and anti-inflammatory NCMs. Singlets were initially identified followed by gating for viable cells using Zombie Violet dye as shown in **Figure 1A**. CMs were identified as CD11b+ Ly6C^hi^ CCR2+ and NCMs were identified as CD11b+ Ly6C^lo^ CD43^hi^ CX3CR1+. **(B)** Flow cytometry gating strategy to identify neutrophils. Singlets were initially identified followed by gating for viable cells using Zombie Violet dye as shown in **Figure 1A**. Neutrophils were identified as Ly6G+ Ly6B.2+. **(C)** CMs in *Prg4^GT/GT^* and *Prg4^+/+^* PLs were higher at 6h. rhPRG4 treatment did not alter CMs in *Prg4^GT/GT^* PLs at 24h. **(D)** NCMs in *Prg4^GT/GT^* and *Prg4^+/+^* PLs were higher at 24h. rhPRG4 treatment increased NCMs in *Prg4^GT/GT^* PLs. **(E)** Neutrophils in *Prg4^GT/GT^* PLs were higher at 24h. rhPRG4 treatment reduced neutrophils in *Prg4^GT/GT^* PLs. **(F)** PL IL-1β levels in *Prg4^GT/GT^* animals at 24h were higher than *Prg4^+/+^* animals and rhPRG4 did not significantly modify IL-1β levels in *Prg4^GT/GT^* animals. **(G)** PL CXCL1 levels in *Prg4^GT/GT^* animals were not different from *Prg4^+/+^* animals at 6h and 24h. rhPRG4 treatment reduced CXCL1 levels in *Prg4^GT/GT^* animals at 24h.

The gating strategies to identify CMs and NCMs are illustrated in **Figure 6A**. Singlets were identified followed by gating for viable cells using Zombie Violet dye as shown in **Figure 1A**. CMs were identified as CD11b+ Ly6C^hi^ CCR2+ while NCMs were identified as CD11b+ Ly6C^lo^ CD43^hi^ CX3CR1+. Neutrophils were identified in lavages using dual positivity for Ly6G and Ly6B.2 (**Figure 6B**). We observed that in *Prg4^GT/GT^* and *Prg4^+/+^* animals, CMs were higher at 6h compared to 24h (*p<0.05*) with no difference between the two genotypes (*p>0.05*) (**Figure 6C**). This was expected since peak inflammation in this model is typically seen at 4h to 6h, and rhPRG4 treatment at 6h did not affect CMs at 24h (*p>0.05*). NCMs were higher at 24h compared to 6h (*p<0.05*) with no difference observed between the two genotypes (*p>0.05*) (**Figure 6D**). rhPRG4 treatment increased NCMs in *Prg4^GT/GT^* peritoneal lavages (*p<0.05*) (**Figure 6D**). At 24h, we also observed more neutrophils in *Prg4^GT/GT^* indicative of unresolved inflammation (*p<0.01*) (**Figure 6E**) and rhPRG4 treatment appeared to reduce neutrophil accumulation at 24h compared to PBS (*p<0.05*) (**Figure 6E**). IL-1β lavage levels were not different between the two genotypes at 6h but higher IL-1β levels were detected at 24h in *Prg4^GT/GT^* animals (*p<0.05*) (**Figure 6F**). While rhPRG4 treatment showed a trend towards a reduction in PL IL-1β at 24h in *Prg4^GT/GT^* animals, this trend was not statistically significant (*p>0.05*), due to a high degree of variability in calculated IL-1β concentrations in specimens from the same experimental group. CXCL1 levels in *Prg4^GT/GT^* PLs decreased with rhPRG4 treatment (*p<0.05*) (**Figure 6G**). Our *in vivo* data support that in the absence of *Prg4* expression, the tissue microenvironment is shifted towards lack of resolution of acute gout inflammation, as indicated by higher neutrophil tissue accumulation and IL-1β levels, and that rhPRG4 promotes effective resolution by increasing the influx of anti-inflammatory NCMs and decreasing CXCL1 and neutrophil tissue accumulation.

## Discussion

In this study, we have documented biologically relevant differences between monocytes from normal and gout patients that advance our understanding of the pathophysiology of acute gout flares. Gout monocytes were phagocytically active under basal conditions, while normal monocytes required a TLR2 stimulatory signal for their phagocytic activation. The phagocytic activation of gout monocytes is potentially related to the systemic inflammatory nature of acute gout which sets it apart from normal monocytes that are not typically exposed to *pro*-inflammatory signals, and thus require priming to be phagocytically active. Gout monocytes showed a greater propensity to uptake antibody-coated beads and urate crystals and the phagocytosis of the latter resulted in enhanced ROS generation and downstream IL-1β production. Phagocytosis is an important cellular process to maintain tissue homeostasis and is a salient component of innate immune defenses against invading microorganisms and foreign particles (33). In phagocytes, internalization of apoptotic cells, microorganisms or insoluble particles is carried out by different receptors, depending on the nature of the ingested particles (33). Antibody-driven phagocytosis of latex beads typically occurs *via* the FCγ receptor (33), while urate crystals are internalized by a receptor-mediated mechanism with CD44, TLR2 and TLR4 being the most prominent mediating receptors (23, 24, 34). IL-1RA and rhPRG4 exhibited interesting differences in modulating normal and gout monocytes under basal and TLR2-stimulated conditions. rhPRG4 has consistently shown superiority in arresting the phagocytic activation of normal and gout monocytes irrespective of their priming status, while the anti-phagocytic effect of IL-1RA was only manifested in normal monocytes following TLR2 ligand priming. Such difference is likely attributed to rhPRG4’s ability to bind multiple cell surface receptors in addition to CD44 *e.g.*, TLR2, TLR4 and other integrins where PRG4 shields monocytes from recognizing extracellular innate immune danger signals (35–37). In contrast, IL-1RA has a specific mechanism of action where it functions through the IL-1 receptor (IL-1R) to inhibit IL-1β mediated signaling. IL-1R and TLR2 belong to the same receptor superfamily and activate similar signaling pathways (38). Furthermore, there is evidence of a crosstalk between TLR2 and IL-1R (39), raising the possibility that IL-1RA inhibits phagocytic activation of normal monocytes by interfering with this crosstalk circuit and thus attenuating the priming effect of TLR2 ligands on monocytes. This conclusion is further supported by IL-1RA’s inability to modulate the phagocytic activity of gout monocytes, where such activity was not dependent on TLR2 ligand priming.

We have also observed that both IL-1RA and rhPRG4 demonstrated dose-dependent reductions in IL-1β release from normal PBMCs with IL-1RA exhibiting higher potency compared to rhPRG4, with an estimated 20-fold potency difference between the two biologics. However, the maximal efficacy of rhPRG4 against normal PBMCs was higher than that of IL-1RA, where rhPRG4 reduced IL-1β release by ~50% compared to 35% with IL-1RA. While gout PBMCs had variable responses to IL-1RA and rhPRG4, both treatments showed biologically meaningful anti-inflammatory effects against gout PBMCs with substantial IL-1β release. In our experiments, both IL-1RA and rhPRG4 were introduced at the TLR2 priming step, and as such most of their anti-inflammatory effect is potentially related to attenuating TLR2 priming of monocytes. In our THP-1 monocyte experiments, rhPRG4 and IL-1RA were introduced at the time monocytes were challenged with urate crystals, allowing for the full TLR2 priming effect to occur. Under these conditions, rhPRG4 suppressed IL-1β to a greater extent, and this suppression was a direct downstream effect of attenuating monocyte phagocytic activation, and ROS generation. Taken together, our data suggests that IL-1RA’s efficacy was dependent on interfering with monocyte priming through the TLR2 pathway while rhPRG4 exerted its biological effect independent of the priming status of monocytes.

The classical NLRP3 inflammasome activation pathway is described as a two-signal pathway with signal one, the priming step mediated by IL-1β or TLR agonists (40). As a result of TLR or IL-1R activation, cellular levels of pro-IL-1β and the inflammasome components increase. The second signal is triggered by DAMPs *e.g.*, urate crystals which results in ROS generation, K+ efflux, activation of the inflammasome and generation of mature IL-1β (40). In monocytes, NLRP3 inflammasome activation is non-classical in nature and several studies have described an alternative inflammasome activation pathway where the priming step is dispensable and the inflammasome activation is gradual in nature with no pyroptotic cell death (41–43). Interestingly, expression of the NLRP3 inflammasome components was not affected by TLR2 priming or urate crystal activation in gout monocytes (44). In our cohort, we have detected higher ROS levels with priming and urate crystal activation in gout monocytes. Gout monocytes displayed increased crystal uptake associated with enhanced ROS generation and IL-1β release, which raises the prospect of a putative mechanism by which higher urate crystal phagocytosis triggers increased ROS burden, which in turn activates the inflammasome. To further examine whether ROS played a causal role in IL-1β release, we treated PBMCs with NAC, which caused a significant reduction in ROS levels in monocytes and a parallel reduction in IL-1β release. This finding lends support to the significant role of urate crystal-generated ROS in IL-1β secretion and provides a rationale for the enhanced IL-1β secretion from gout PBMCs. Our THP-1 monocyte experiments further support that rhPRG4’s anti-inflammatory effect is linked to its ability to reduce ROS generation in monocytes. NLRP3 protein levels remained unchanged in THP-1 monocytes following priming and urate crystal activation, consistent with our previous observation (26). In our experiments, transcriptional priming of pro-IL-1β was observed along with an increase in caspase-1 activity, which combined explain IL-1β release by THP-1 monocytes. rhPRG4’s anti-inflammatory effect was mediated by attenuation of pro-IL-1β transcriptional priming and normalization of caspase-1 activity and therefore, its effect is a combination of inflammasome activation dependent and independent effects. The blunted priming step with rhPRG4 treatment is likely due to the latter’s ability to attenuate nuclear factor kappa B (NFκB) signaling axis downstream of TLR2 activation (23, 35). We observed that PP2A activity in monocytes was impaired with TLR2 priming and urate crystals. While the underlying mechanism remains unknown, one potential explanation is the inactivation of PP2A by ROS, generated following urate crystal uptake by monocytes (45). PP2A is a cytosolic serine/threonine phosphatase that is expressed in multiple organs and in the immune system (46). In monocytes and macrophages, PP2A functions to regulate the innate immune response by TLR ligands including urate crystals. Conditional knockout of the PP2A catalytic subunit enhanced tumor necrosis factor alpha (TNF-a) expression in lipopolysaccharide (LPS)-stimulated murine macrophages (47). Using THP-1 monocytes, we have also shown that PP2A catalytic subunit knockdown exacerbated IL-1β secretion in response to urate crystals (26). CD44 receptor engagement can activate various signaling pathways dependent on the nature of the ligand and the specific cell type (48). In our study, rhPRG4 activated PP2A, potentially due to its binding CD44, which in turn contributed to its anti-inflammatory efficacy. Using okadaic acid, we showed that PP2A inhibition reverses rhPRG4’s effect, magnifies pro-IL-1β production and increases caspase-1 activity in monocytes without altering NLRP3 levels. While okadaic acid was shown to also inhibit protein phosphatase-1 (PP-1), okadaic acid’s potency against PP2A is more than 50 times higher than that of PP-1 (30). Considering the concentration of okadaic acid in our experiments, it is reasonable to expect that its effect was solely due to PP2A inhibition. Our data indicate that the biological effect of rhPRG4 in urate crystal stimulated monocytes was partially mediated by PP2A activation resulting in attenuating pro-IL-1β production and caspase-1 activity and thus suppressing mature IL-1β secretion.

In acute gout flares, tissue influx of immune cells e.g., monocytes and neutrophils is temporally regulated, where these cells play important roles in orchestrating the onset, progression and resolution of inflammation (7). CMs are *pro*-inflammatory and are characterized by high expression levels of Ly6C and predominantly express the pro-recruitment receptor CCR2 (27, 49). CMs extravasate into inflamed tissues where they differentiate into inflammatory M1 macrophages (49). In contrast, NCMs aid in wound healing, clearance of debris and preferentially differentiate into the alternatively activated wound-healing macrophages (50). In the peritoneal model of acute gout, an initial surge of CMs and neutrophils was observed at 6h, coinciding with peak inflammation, followed by delayed tissue influx of NCMs coinciding with resolution of inflammation. We did not detect meaningful differences in the numbers of recruited CMs or neutrophils between *Prg4* null and competent animals, which indicates that the inflammatory response in this model is not dependent on *Prg4* expression. However, the absence of Prg4’s biological function was associated with accumulation of viable neutrophils and hence inadequate resolution of inflammation. This assumption is buttressed by detectable IL-1β at 24h in Prg4 deficient mice but not Prg4 competent counterparts. This is especially true since the introduction of exogenous PRG4 had a resolving effect, demonstrated by enhanced NCM tissue influx and proper clearance of neutrophils. Our CXCL1 data are also in line with less neutrophils recovered from rhPRG4-treated animals, since CXCL1 is a neutrophil chemoattractant, where its reduced levels may have contributed to resolution of inflammation (7). Our *in vivo* observations should be rationalized in the context of our recent discoveries regarding PRG4’s role in tissue macrophage homeostasis and function. We have recently shown that in *Prg4^GT/GT^* mice, the synovial microenvironment skews the balance of resident macrophages towards a pro-inflammatory M1 phenotype with age-dependent progressive decline in the numbers of anti-inflammatory wound-healing macrophages (51). Furthermore, macrophages from *Prg4^GT/GT^* animals had reduced anti-inflammatory interleukin-10 (IL-10) expression at baseline and in response to TLR2 ligand stimulation (51). Extending these findings to our peritoneal model, it is plausible to expect that the impaired expression of IL-10 in *Prg4^GT/GT^* mice contributed to the lack of adequate resolution of inflammation (27). Furthermore, the re-introduction of PRG4 may have blunted the overall pro-inflammatory tissue microenvironment in *Prg4^GT/GT^* animals (51), therefore allowing for more NCM influx and clearance of neutrophils.

PRG4 is a mucinous glycoprotein with the highest expression seen in the synovial joint, bone and liver (52). PRG4’s protein core is 1,404 amino acid long with *N* and C termini globular domains, and a central mucin domain that is heavily glycosylated *via O*-linked β (1-3) Gal-GalNac oligosaccharides (53). In the articular joint, PRG4 fulfills a multifaceted role as a cartilage boundary lubricant and a homeostatic regulator of the synovium (54–56). PRG4 has anti-inflammatory activity, mediated by its CD44 interaction, resulting in attenuating NFκB nuclear translocation (55, 57). This activity may explain rhPRG4’s disease-modifying activity seen in pre-clinical osteoarthritis models (58, 59). The anti-inflammatory activity of rhPRG4 was also demonstrated in an *in vitro* sepsis model, where rhPRG4 at 50 or 100μg/mL reduced interleukin-6 (IL-6) secretion from murine and human endothelial cells (60). rhPRG4 was also shown to reduce cytokine and chemokine secretions from immortalized corneal epithelial cells at 300μg/mL *in vitro* and inflammation in an experimental dry eye disease model (61). rhPRG4 reduced neuroinflammation and influx of monocytes in a rat model of traumatic brain injury, mediated by its regulation of the ERK1/2 pathway, downstream of CD44 and TLR2/4 engagements (62). Our current study extends the anti-inflammatory activity of rhPRG4 to controlling IL-1β secretion by PBMCs from patients with acute gout flares, where the efficacious rhPRG4 concentration was in the range of what was reported as efficacious in *in vitro* models of human synoviocytes, macrophages, endothelial and corneal epithelial cells. Our study was limited by the sparse clinical characteristics data collected on our acute gout patient cohort, *e.g.*, comorbidities and medication history. The use of oral colchicine probably reduced the amounts of IL-1β secreted from gout PBMCs. In addition, the conclusions of our study are limited by the small size of the cohort, as we plan to investigate the efficacy of rhPRG4 using monocytes from a large gout cohort.

In summary, our study is the first to show experimental evidence supporting the anti-inflammatory activity of rhPRG4 using PBMCs from patients with acute gout flares. rhPRG4 reduced phagocytic activation of gout monocytes both under basal and primed conditions and this reduction resulted in a downstream attenuation of IL-1β secretion, and rhPRG4’s efficacy was equivalent to a clinically approved biologic, IL-1RA. The anti-inflammatory activity of rhPRG4 was mediated by its ability to reduce ROS generation, IL-1β expression and caspase-1 activity, and hence conversion of pro-IL-1β to mature IL-1β, in human monocytes following TLR2 ligand priming and urate crystal activation. rhPRG4’s effect was a combined result of inhibiting phagocytic activation of monocytes and cytosolic PP2A activity enhancement. PRG4 may also play a significant role in mediating the resolution of acute gout inflammation. We conclude that rhPRG4 is a potential new therapeutic for treatment of acute gout flares.

## Data Availability Statement

The raw data supporting the conclusions of this article will be made available by the authors, without undue reservation.

## Ethics Statement

The studies involving human participants were reviewed and approved by Institutional Review Board (IRB) at The Rhode Island and Mariam Hospitals. The patients/participants provided their written informed consent to participate in this study. The animal study was reviewed and approved by IACUC Committee, Chapman University.

## Author Contributions

Authors SE, GJ, and KE conceived the study. Authors RC and GJ enrolled patients, collected blood and isolated PBMCs, and participated in data analysis and interpretation of results. Authors SE, MQ, and KE carried out experiments and participated in analysis of data. Author TS provided reagents and critical interpretation of results. All authors have participated in drafting and critical evaluation of the manuscript. All authors have read and approved the final version of the manuscript.

## Funding

This work is supported by R01AR067748 to KE and GJ.

## Conflict of Interest

Author GJ authored patents on rhPRG4 and holds equity in Lubris LLC, MA, USA. Author TS authored patents on rhPRG4, is a paid consultant for Lubris LLC, MA, USA and holds equity in Lubris LLC, MA, USA. Author KE authored patents on rhPRG4.

The remaining authors declare that the research was conducted in the absence of any commercial or financial relationships that could be construed as a potential conflict of interest.

## Publisher’s Note

All claims expressed in this article are solely those of the authors and do not necessarily represent those of their affiliated organizations, or those of the publisher, the editors and the reviewers. Any product that may be evaluated in this article, or claim that may be made by its manufacturer, is not guaranteed or endorsed by the publisher.

## References

[1] Dehlin M Jacobsson L Roddy E. Global Epidemiology of Gout: Prevalence, Incidence, Treatment Patterns and Risk Factors. Nat Rev Rheumatol (2020) 16:380–90. doi: 10.1038/s41584-020-0441-1 32541923

[2] Chen-Xu M Yokose C Rai SK Pillinger MH Choi HK. Contemporary Prevalence of Gout and Hyperuricemia in the United States and Decadal Trends: The National Health and Nutrition Examination Survey, 2007-2016. Arthritis Rheumatol (2019) 71:991–9. doi: 10.1002/art.40807 PMC6536335 30618180

[3] Chen Y Tang Z Huang Z Zhou W Li Z Li X. The Prevalence of Gout in Mainland China From 2000 to 2016: A Systematic Review and Meta-Analysis. J Public Health (2017) 25:521–9. doi: 10.1007/s10389-017-0812-5

[4] Wandell P Carlsson AC Ljunggren G. Gout and Its Comorbidities in the Total Population of Stockholm. Prev Med (2015) 81:387–91. doi: 10.1016/j.ypmed.2015.10.003 26500085

[5] Kinge JM Knudsen AK Skirbekk V Vollset SE. Musculoskeletal Disorders in Norway: Prevalence of Chronicity and Use of Primary and Specialist Health Care Services. BMC Musculoskelet Disord (2015) 16:75. doi: 10.1186/s12891-015-0536-z 25887763 PMC4392859

[6] Elfishawi M Zleik N Kvergic Z Michet CJ Crowson CS Matteson EL. The Rising Incidence of Gout and the Increasing Burden of Comorbidities: A Population-Based Study Over 20 Years. J Rheumatol (2018) 45:574–9. doi: 10.3899/jrheum.170806 PMC5880714 29247151

[7] Dalbeth N Choi HK Joosten LA Khanna PP Matsuo H Perez-Ruiz F. Gout. Nat Rev Dis Primers (2019) 5:69. doi: 10.1038/s41572-019-0115-y 31558729

[8] Taylor WJ Fransen J Jansen TL Dalbeth N Schumacher HR Brown M. Study for Updated Gout Classification Criteria (SUGAR): Identification of Features to Classify Gout. Arthritis Care Res (Hoboken) (2015) 67:1304–15. doi: 10.1002/acr.22585 PMC4573373 25777045

[9] Khanna D Khanna PP Fitzgerald JD Singh MK Bae S Neogi T. 2012 American College of Rheumatology Guidelines for Management of Gout Part II: Therapy and Anti-Inflammatory Prophylaxis of Acute Gouty Arthritis. Arthritis Care Res (Hoboken) (2012) 64:1447–61. doi: 10.1002/acr.21773 PMC3662546 23024029

[10] Richette P Doherty M Pascual E Barskova V Becce F Castaneda-Sanabria J. 2016 Updated EULAR Evidence-Based Recommendations for the Management of Gout. Ann Rheum Dis (2017) 76:29–42. doi: 10.1136/annrheumdis-2016-209707 27457514

[11] Janssens HJ Lucassen PL Van de Laar FA Janssen M Van de Lisdonk EH. Systemic Corticosteroids for Acute Gout. Cochrane Database Syst Rev (2008) 2:CD005521. doi: 10.1002/14651858.CD005521.pub2 PMC8276233 18425920

[12] Van Echted I Wechalekar MD Schlesinger N Buchbinder R Aletaha D. Colchicine for Acute Gout. Cochrane Database Syst Rev (2014) 8:CD006190. doi: 10.1002/14651858.CD006190.pub2 25123076

[13] So A Dumusc A Nasi S. The Role of IL-1 in Gout: From Bench to Bedside. Rheumatology (2018) 57:i12–9. doi: 10.1093/rheumatology/kex449 29272514

[14] Schlesinger N. Anti-Interleukin-1 Therapy in the Management of Gout. Curr Rheumatol Rep (2014) 16:398. doi: 10.1007/s11926-013-0398-z 24407823

[15] Martin WJ Walton M Harper J. Resident Macrophages Initiating and Driving Inflammation in a Monosodium Urate Monohydrate Crystal-Induced Murine Peritoneal Model of Acute Gout. Arthritis Rheum (2009) 60:281–9. doi: 10.1002/art.24185 19116939

[16] Busso N So A. Mechanisms of Inflammation in Gout. Arthritis Res Ther (2010) 12:206. doi: 10.1186/ar2952 20441605 PMC2888190

[17] Yang G Yeon SH Lee HE Kang HC Cho YY Lee HS. Suppression of NLRP3 Inflammasome by Oral Treatment With Sulforaphane Alleviates Acute Gouty Inflammation. Rheumatology (2018) 57:727–36. doi: 10.1093/rheumatology/kex499 29340626

[18] Giamarellos-Bourboulis EJ Mouktaroudi M Boder E van der Ven J Kullberg B-J Netea MG. Crystals of Monosodium Urate Monohydrate Enhance Lipopolysaccharide-Induced Release of Interleukin-1 Beta by Mononuclear Cells Through a Caspase-1 Mediated Process. Ann Rheum Dis (2009) 68:273–8. doi: 10.1136/ard.2007.082222 18390571

[19] Joosten LA Netea MG Mylona E Koenders MI Malireddi RKS Oosting M. Engagement of Fatty Acids With Toll-Like Receptor 2 Drives Interleukin-1b Production *via* the ASC/caspase 1 Pathway in Monosodium Urate Monohydrate Crystal-Induced Gouty Arthritis. Arthritis Rheum (2010) 62:3237–48. doi: 10.1002/art.27667 PMC2970687 20662061

[20] Martin WJ Shaw O Liu X Steiger S Harper JL. Monosodium Urate Monohydrate Crystal-Recruited Noninflammatory Monocytes Differentiate Into M1-Like Proinflammatory Macrophages in a Peritoneal Murine Model of Gout. Arthritis Rheum (2011) 63:1322–32. doi: 10.1002/art.30249 21538316

[21] Mylona EE Mouktaroudi M Crisan TO Makri S Pistiki A Georgitsi M. Enhanced Interleukin-1β Production of PBMCs From Patients With Gout After Stimulation With Toll-Like Receptor-2 Ligands and Urate Crystals. Arthritis Res Ther (2012) 14:R158. doi: 10.1186/ar3898 22762240 PMC3580550

[22] Crisan TO Cleophas MCP Novakovic B Erler K van de Veerdonk FL Stunnenberg HG. Uric Acid Priming in Human Monocytes Is Driven by the AKT-PRAS40 Autophagy Pathway. Proc Natl Acad Sci USA (2017) 114:5485–90. doi: 10.1073/pnas.1620910114 PMC5448210 28484006

[23] Qadri M Jay GD Zhang LX Wong W Reginato AM Sun C. Recombinant Human Proteoglycan-4 Reduces Phagocytosis of Urate Crystals and Downstream Nuclear Factor Kappa B and Inflammasome Activation and Production of Cytokines and Chemokines in Human and Murine Macrophages. Arthritis Res Ther (2018) 20:192. doi: 10.1186/s13075-018-1693-x 30157934 PMC6116363

[24] Bousoik E Qadri M Elsaid KA. CD44 Receptor Mediates Crystal Phagocytosis by Macrophages and Regulates Inflammation in a Murine Peritoneal Model of Acute Gout. Sci Rep (2020) 10:5748. doi: 10.1038/s41598-020-62727-z 32238827 PMC7113258

[25] Zoghbi K Bousoik E Parang K Elsaid KA. Design and Biological Evaluation of Colchicine-CD44-Targeted Peptide Conjugate in an *In Vitro* Model of Crystal Induced Inflammation. Molecules (2020) 25:45. doi: 10.3390/molecules25010046 PMC6982808 31877739

[26] Qadri M Elsayed S Elsaid KA. Fingolimod Phosphate (FTY720-P) Activates Protein Phosphatase 2A in Human Monocytes and Inhibits Monosodium Urate Crystal-Induced IL-1β Production. J Pharmacol Exp Ther (2021) 376:222–30. doi: 10.1124/jpet.120.000321 PMC7873533 33239408

[27] Narasimhan PB Marcovecchio P Hamers AA Hedrick CC. Nonclassical Monocytes in Health and Disease. Ann Rev Immunol (2019) 37:439–56. doi: 10.1146/annurev-immunol-042617-053119 31026415

[28] Grievink HW Luisman T Kluft C Moerland M Malone K. Comparison of Three Isolation Techniques for Human Peripheral Blood Mononuclear Cells: Cell Recovery and Viability, Population Composition, and Cell Functionality. Biopreserv Biobank (2016) 14:410–5. doi: 10.1089/bio.2015.0104 27104742

[29] Flynn CM Garbers Y Lokau J Wesch D Schulte DM Laudes M. Activation of Toll-Like Receptor 2 (TLR2) Induces Interleukin-6 Trans-Signaling. Sci Rep (2019) 9:7306. doi: 10.1038/s41598-019-43617-5 31086276 PMC6513869

[30] Valdiglesias V Prego-Faraldo MV Pasaro E Mendez J Laffon B. Okadaic Acid: More Than a Diarrheic Toxin. Mar Drugs (2013) 11:4328–49. doi: 10.3390/md11114328 PMC3853731 24184795

[31] Hill A Waller KA Cui Y Allen JM Smits P Zhang LX. Lubricin Restoration in a Mouse Model of Congenital Deficiency. Arthritis Rheumatol (2015) 67(11):3070–81. doi: 10.1002/art.39276 PMC4626303 26216721

[32] Samson ML Morrison S Masala N Sullivan BD Sullivan DA Sheardown H. Characterization of Full-Length Recombinant Human Proteoglycan 4 as an Ocular Surface Boundary Lubricant. Exp Eye Res (2014) 127:14–9. doi: 10.1016/j.exer.2014.06.015 24997456

[33] Uribe-Querol E Rosales C. Phagocytosis: Our Current Understanding of a Universal Biological Process. Front Immunol (2020) 11:1066. doi: 10.3389/fimmu.2020.01066 32582172 PMC7280488

[34] Liu-Bryan R Scott P Sydlaske A Rose DM Terkeltaub R. Innate Immunity Conferred by Toll-Like Receptors 2 and 4 and Myeloid Differentiation Factor 88 Expression Is Pivotal to Monosodium Urate Monohydrate Crystal-Induced Inflammation. Arthritis Rheum (2005) 52:2936–46. doi: 10.1002/art.21238 16142712

[35] Alquraini A Garguilo S D’Souza G Zhang LX Schmidt TA Jay GD. The Interaction of Lubricin/Proteoglycan 4 (PRG4) With Toll-Like Receptors 2 and 4: An Anti-Inflammatory Role of PRG4 in Synovial Fluid. Arthritis Res Ther (2015) 17:353. doi: 10.1186/s13075-015-0877-x 26643105 PMC4672561

[36] Iqbal SM Leonard C Regmi SC De Rantere D Tailor P Ren G. Lubricin/proteoglycan 4 Binds to and Regulates the Activity of Toll-Like Receptors. vitro Sci Rep (2016) 6:18910. doi: 10.1038/srep18910 26752378 PMC4707532

[37] Jin C Ekwell AK Bylund J Bjorkman L Estrella RP Whitelock JM. Human Synovial Lubricin Expresses Sialyl Lewis X Determinant and has L-Selectin Ligand Activity. J Biol Chem (2012) 287:35922–33. doi: 10.1074/jbc.M112.363119 PMC3476260 22930755

[38] O’Neil LA. The Interleukin-1 Receptor/Toll-Like Receptor Superfamily: 10 Years of Progress. Immunol Rev (2008) 226:10–8. doi: 10.1111/j.1600-065X.2008.00701.x 19161412

[39] Kawai T Akira S. Toll-Like Receptors and Their Crosstalk With Other Innate Receptors in Infection and Immunity. Immunity (2011) 34:637–50. doi: 10.1016/j.immuni.2011.05.006 21616434

[40] Yang Y Wang H Koudair M Song H Shi F. Recent Advances in the Mechanisms of NLRP3 Inflammasome Activation and Its Inhibitors. Cell Death Dis (2019) 10:128. doi: 10.1038/s41419-019-1413-8 30755589 PMC6372664

[41] Crisan TO Cleophas MC Oosting M Lemmers H Dijkstra HT Netea MG. Soluble Uric Acid Primes TLR-Induced Proinflammatory Cytokine Production by Human Primary Cells *via* Inhibition of IL-1ra. Ann Rheum Dis (2016) 75:755–62. doi: 10.1136/annrheumdis-2014-206564 25649144

[42] Gaidt M Ebert TS Chauhan D Schmidt T Schmid-Burgk JL Rapino F. Human Monocytes Engage an Alternative Inflammasome Pathway. Immunity (2016) 44:833–46. doi: 10.1016/j.immuni.2016.01.012 27037191

[43] Gritsenko A Yu S Martin-Sanchez F Diaz-del-Olmo I Nichols EM Davis DM. Priming Is Dispensable for NLRP3 Inflammasome Activation in Human Monocytes *In Vitro* . Front Immunol (2020) 11:565924. doi: 10.3389/fimmu.2020.565924 33101286 PMC7555430

[44] Alberts BM Bruce C Basnayake K Ghezzi P Davies KA Mullen M. Secretion of IL-1β From Monocytes in Gout Is Redox Independent. Front Immunol (2019) 10:70. doi: 10.3389/fimmu.2019.00070 30761138 PMC6361747

[45] Raman D Pervaiz S. Redox Inhibition of Protein Phosphatase Pp2A: Potential Implications in Oncogenesis and Its Progression. Redox Biol (2019) 27:101105. doi: 10.1016/j.redox.2019.101105 30686777 PMC6859563

[46] Reynhaut S Janssens V. Physiologic Functions of PP2A: Lessons From Genetically Modified Mice. Biochem Biophys Acta Mol Cell Res (2019) 1866:31–50. doi: 10.1016/j.bbamcr.2018.07.010 30030003

[47] Sun L Pham TT Cornell TT McDonough KL McHugh WM Blatt NB. Myeloid-Specific Gene Deletion of Protein Phosphatase 2A Magnifies MyD88- and TRIF-Dependent Inflammation Following Endotoxin Challenge. J Immunol (2017) 198:404–16. doi: 10.4049/jimmunol.1600221 PMC5173401 27872207

[48] Jordan AR Racine RR Henning MJ Lokeshwar VB. The Role of CD44 in Disease Pathophysiology and Targeted Treatment. Front Immunol (2015) 6:182. doi: 10.3389/fimmu.2015.00182 25954275 PMC4404944

[49] Cormican S Griffin M. Human Monocyte Subset Distinctions and Function: Insights From Gene Expression Analysis. Front Immunol (2020) 11:1070. doi: 10.3389/fimmu.2020.01070 32582174 PMC7287163

[50] Olingy CE San Emeterio CL Ogle ME Krieger JR Bruce AC Pfau DD. Non-Classical Monocytes Are Biased Progenitors of Wound Healing Macrophages During Soft Tissue Injury. Sci Rep (2017) 7:447. doi: 10.1038/s41598-017-00477-1 28348370 PMC5428475

[51] Qadri M Zhang LX Jay GD Schmidt TA Totonchy J Elsaid KA. Proteoglycan-4 Is an Essential Regulator of Synovial Macrophage Polarization and Inflammatory Macrophage Joint Infiltration. Arthritis Res Ther (2021) 23:241. doi: 10.1186/s13075-021-02621-9 34521469 PMC8439011

[52] Novince CM Koh AJ Michalski MN Marchesan JT Wang J Jung Y. Proteoglycan 4, a Novel Immunomodulatory Factor, Regulates Parathyroid Hormone Actions on Hematopoietic Cells. Am J Pathol (2011) 179:2431–42. doi: 10.1016/j.ajpath.2011.07.032 PMC3204095 21939632

[53] Jay GD Tantravahi U Britt DE Barrach HJ Cha CJ. Homology of Lubricin and Superficial Zone Protein (SZP): Products of Megakaryocyte Stimulating Factor (MSF) Gene Expression by Human Synovial Fibroblasts and Articular Chondrocytes Localized to Chromosome 1q25. J Orthop Res (2001) 194:677–87. doi: 10.1016/S0736-0266(00)00040-1 11518279

[54] Jay GD Waller KA. The Biology of Lubricin: Near Frictionless Joint Motion. Matrix Biol (2014) 39:17–24. doi: 10.1016/j.matbio.2014.08.008 25172828

[55] Alquraini A Jamal M Zhang L Schmidt TA Jay GD Elsaid KA. The Autocrine Role of Proteoglycan-4 (PRG4) in Modulating Osteoarthritic Synoviocyte Proliferation and Expression of Matrix Degrading Enzymes. Arthritis Res Ther (2017) 19:89. doi: 10.1186/s13075-017-1301-5 28482921 PMC5423025

[56] Qadri M Jay GD Zhang LX Richendrfer H Schmidt TA Elsaid KA. Proteoglycan-4 Regulates Fibroblast to Myofibroblast Transition and Expression of Fibrotic Genes in the Synovium. Arthritis Res Ther (2020) 22:113. doi: 10.1186/s13075-020-02207-x 32404156 PMC7222325

[57] Al-Sharif A Jamal M Zhang LX Larson K Schmidt TA Jay GD. Lubricin/Proteoglycan 4 Binding to CD44 Receptor: A Mechanism of the Suppression of Proinflammatory Cytokine-Induced Synoviocyte Proliferation by Lubricin. Arthritis Rheumatol (2015) 67:1503–13. doi: 10.1002/art.39087 PMC4446227 25708025

[58] Jay GD Fleming BC Watkins BA McHugh KA Anderson SC Zhang LX. Prevention of Cartilage Degeneration and Restoration of Chondroprotection by Lubricin Tribosupplementation in the Rat Following Anterior Cruciate Ligament Transection. Arthritis Rheum (2010) 62:2382–91. doi: 10.1002/art.27550 PMC2921027 20506144

[59] Waller KA Chin KE Jay GD Zhang LX Teeple E McAllister S. Intra-Articular Recombinant Human Proteoglycan-4 Mitigates Cartilage Damage After Destabilization of the Medial Meniscus in the Yucatan Minipig. Am J Sports Med (2017) 45:1512–21. doi: 10.1177/0363546516686965 PMC5453820 28129516

[60] Richendrfer HA Levy M Elsaid KA Schmidt TA Zhang L Cabezas R. Recombinant Human Proteoglycan-4 Mediates Interleukin-6 Response in Both Human and Mouse Endothelial Cells Induced Into a Sepsis Phenotype. Crit Care Explor (2020) 2:e0126. doi: 10.1097/CCE.0000000000000126 32695993 PMC7314356

[61] Menon NG Goyal R Lema C Woods PS Tanguay AP Morin AA. Proteoglycan 4 (PRG4) Expression and Function in Dry Eye Associated Inflammation. Exp Eye Res (2021) 208:108628. doi: 10.1016/j.exer.2021.108628 34048779 PMC8491169

[62] Bennett M Chin A Lee HJ Cestero EM Strazielle N Ghersi-Egea JF. Proteoglycan 4 Reduces Neuroinflammation and Protects the Blood-Brain Barrier After Traumatic Brain Injury. J Neurotrauma (2021) 38:385–98. doi: 10.1089/neu.2020.7229 PMC7875610 32940130

